# The MATTERS Trial: Safety and Tolerability of Whole-Body Hyperthermia at 41.50°C in Combination with Chemotherapy in Metastatic Cancer Patients

**DOI:** 10.1158/2767-9764.CRC-25-0660

**Published:** 2026-02-04

**Authors:** Luigi Brancato, Ivana Gorbaslieva, Oleg Rudenko, Tine Logghe, Eke van Zwol, Gaëlle Boulet, Laurent Van den Bossche, Johan Van den Bossche, Marc Peeters, Thiery Chapelle, Vera Saldien, Peter Vueghs, Timon Vandamme, Rowan Dankerlui, Dirk Ysebaert, Johannes Bogers

**Affiliations:** 1 ElmediX NV, Leuven, Belgium.; 2 Faculty of Medicine and Health Sciences, University of Antwerp, Antwerp, Belgium.; 3 Department of Hepatobiliary, Transplant and Endocrine Surgery, University Hospital Antwerp, Antwerp, Belgium.; 4 Department of Anesthesiology, University Hospital Antwerp, Antwerp, Belgium.; 5 Department of Anesthesiology, AZ Vitaz, Sint-Niklaas, Belgium.; 6 Center of Oncological Research (CORE), Faculty of Medicine and Health Sciences, University of Antwerp, Antwerp, Belgium.

## Abstract

**Purpose:**

Whole-body hyperthermia (WBHT) is a promising therapy for advanced malignancies, including metastatic pancreatic ductal adenocarcinoma (PDAC). This first-in-human study evaluated the safety and tolerability of repeated WBHT sessions at 41.50°C for various treatment durations and in combination with standard-of-care chemotherapy regimens.

**Patients and Methods:**

Twelve patients with advanced solid tumors, primarily metastatic PDAC, completed the study. WBHT was administered using an innovative medical device (TempoCure, ElmediX) as monotherapy or with standard-of-care chemotherapy in escalating durations of 2, 4, and 6 hours, in four cohorts. Safety was assessed through adverse event (AE) monitoring, serious AE (SAE) reporting, and comprehensive clinical and laboratory evaluations up to a 10-week follow-up. Tolerability was evaluated according to whether patients were able to complete all planned WBHT cycles, with or without concomitant chemotherapy, without the need for dose modifications or treatment interruptions.

**Results:**

The WBHT treatment was well tolerated, with most patients completing scheduled treatments despite advanced disease. The most frequent AEs were fatigue, hypokalemia, nausea, and diarrhea. Initial decubitus ulcers led to improved patient handling protocols. No clear increase in AE or SAE incidence was associated with longer WBHT durations or chemotherapy combination. The trend toward fewer AEs in later treatments likely reflects a procedural learning curve.

**Conclusions:**

WBHT administered with the TempoCure system at 41.50°C for 2, 4, and 6 hours is safe and tolerable alone or in combination with chemotherapy, in patients with advanced cancer refractory to prior treatments. These findings support further investigation in upcoming randomized trials designed to evaluate efficacy in metastatic PDAC.

**Significance:**

The MATTERS trial demonstrates that WBHT at 41.50°C, delivered with the TempoCure device, is a safe and well-tolerated treatment for advanced malignancies, particularly in patients with refractory metastatic PDAC. These findings support the integration of WBHT with standard chemotherapy regimens, paving the way for future efficacy trials aimed at improving therapeutic outcomes in challenging cancer populations.

## Introduction

Facing the persistent lack of effective treatments for a number of solid cancers, various forms of hyperthermia therapy (HT) are being increasingly researched. Systemic HT, or whole-body hyperthermia (WBHT), offers a unique advantage for patients with disseminated malignancies, unlike local or regional approaches that target confined tumor sites (1, 2). When combined with radiotherapy or chemotherapy, HT has demonstrated synergistic effects that enhance the efficacy of conventional treatments (3). Proposed mechanisms include direct cytotoxicity, immune modulation, alteration of tumor vasculature and stromal architecture to enhance drug delivery and immune cell infiltration, and sensitization of cancer cells through reversal of drug resistance (3, 4).

Pancreatic ductal adenocarcinoma (PDAC), which accounts for ≥85% of pancreatic cancers, is among the most aggressive and treatment-resistant malignancies. More than 80% of cases are diagnosed at an advanced stage, and only 15% to 20% of patients are eligible for surgical resection (5, 6). For the majority of the patients, chemotherapy remains the primary treatment option. Guideline regimens, selected based on performance status, include FOLFIRINOX, modified FOLFIRINOX, gemcitabine/nab-paclitaxel, NALIRIFOX, or gemcitabine monotherapy (7). Despite advances in systemic therapy and supportive care, outcomes remain poor: the 5-year relative survival rate is only 11% across all stages and only 3% for metastatic disease, with minimal improvement over the past four decades (6). The dismal prognosis is driven by late-stage diagnosis, rapid progression, and the intrinsic and acquired resistance of PDAC to existing therapies (8).

Emerging evidence supports HT’s potential as an adjunct modality in the treatment of advanced malignancies such as PDAC (9–13).

**Figure 1.**
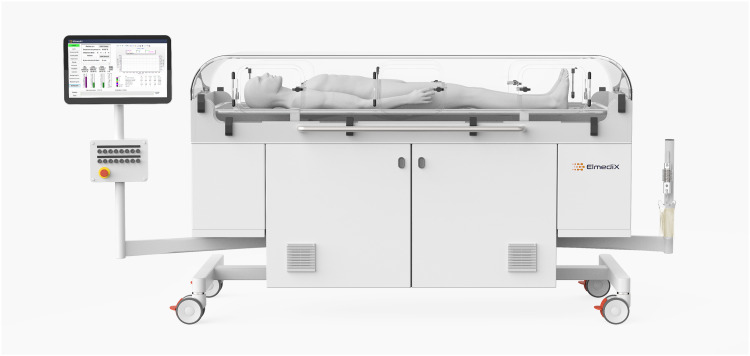
The TempoCure system (ElmediX, Belgium) used for WBHT treatments in the MATTERS study. This innovative medical device enables precise regulation of core body temperature through a closed-loop control system, ensuring patient safety and comfort during treatment.

A systematic review by van der Horst and colleagues (14) evaluated the clinical outcomes of HT combined with chemotherapy and/or radiotherapy in locally advanced or metastatic pancreatic cancer. Among 14 studies involving 395 patients (248 treated with HT, among which only 20 were treated with systemic hyperthermia), those receiving HT showed longer median overall survival (11.7 months vs. 5.6 months) and a higher overall response rate (43.9% vs. 35.3%) compared with controls. However, the authors noted methodologic limitations and emphasized the need for rigorously designed prospective trials to confirm the clinical value of HT in PDAC. Although WBHT at fever-range temperatures has shown feasibility, advancing to higher therapeutic temperatures necessitates significant technical innovations, including precise temperature regulation and reliable heating system performance. These requirements have driven the development of the TempoCure medical device (Fig. 1). TempoCure represents a significant advancement in WBHT technology, overcoming prior limitations in achieving precise spatial and temporal temperature control, which is essential for safe and effective treatment at elevated thermal doses. The selected induced temperature of 41.50°C was intended to enhance antigen presentation and activate innate and adaptive immune cells. It aims to promote tumor infiltration while addressing genome instability and mutations by impairing DNA repair pathways and increasing tumor vulnerability. This approach is designed to avoid sublethal or irreversible tissue damage, particularly in sensitive organs such as the brain, liver, muscles, and skin, which can occur above 42°C (13, 15–17).

Before advancing to randomized efficacy studies, it is essential to establish the safety, tolerability, and technical feasibility of WBHT for 2, 4, and 6 hours at 41.50°C in patients with cancer. Key factors influencing outcomes include thermal dose (the combination of temperature and duration), heating system reliability, spatial and temporal temperature control, and compatibility with standard oncologic treatments. Achieving a thermal dose that maximizes therapeutic benefit, without dose-limiting toxicity to normal tissues, is a central challenge, one addressed through the precise temperature regulation capabilities of the innovative device used in this study (TempoCure).

To address these foundational considerations, we initiated MATTERS, a first-in-human (FIH), single-center safety study of WBHT in combination with gemcitabine with focus on patients with metastatic PDAC. The MATTERS protocol uses a dose-escalation design based on a fixed-target core body temperature of 41.50°C and variable WBHT treatment durations to assess the safety and tolerability of increasing thermal doses. It also evaluates the feasibility of combining WBHT with standard-of-care chemotherapy regimens in alignment with the current National Comprehensive Cancer Network (NCCN) treatment guidelines.

By assessing safety, tolerability, and both technical and clinical feasibility in this early clinical setting, MATTERS aims to generate essential data to guide the design of future efficacy-focused trials in metastatic PDAC. The selected temperature of 41.50°C and treatment durations were based on available scientific literature and preclinical evidence, while remaining within physiologically tolerable limits (18). The study builds upon robust translational data from large-animal models, including healthy pigs and tumor-bearing dogs, which together established the biological rationale, technical viability, and preliminary safety of the WBHT platform (19, 20).

## Patients and Methods

The MATTERS study evaluated the safety and tolerability of WBHT in patients with advanced cancers, including metastatic PDAC. This study included four sequential cohorts (A1, A2, B, and C), each designed to evaluate WBHT safety and tolerability under varying conditions, as detailed in the Study Design section below.

### Intervention

The intervention protocol integrated four key components: WBHT, simultaneous temperature monitoring at various body locations, general anesthesia, and chemotherapy. Each element was meticulously coordinated to ensure patient safety, comfort, and adherence to standard oncological care. This protocol was designed based on preclinical research using data obtained from *in vitro* and *in vivo* models that guided critical refinements in anesthetic agent selection, fluid management, and monitoring strategies to enhance safety and maintain physiologic stability during WBHT treatment.

WBHT treatments were conducted at the University Hospital of Antwerp, Belgium, using the TempoCure system (ElmediX; Fig. 1). This device was engineered to deliver WBHT under tightly controlled conditions by employing a closed-loop control system to precisely regulate the patient’s core temperature. The target temperature for all sessions was set at 41.50°C.

Temperature monitoring was performed continuously and at multiple anatomic sites using the TempoSensor flexible probes (ElmediX). In the absence of specific European Medicines Agency guidance on thermal monitoring for hyperthermia-based interventions, we followed the more detailed recommendations outlined in the FDA’s draft guidance (Evaluation of Thermal Effects of Medical Devices that Produce Tissue Heating and/or Cooling; ref. 21). The primary control input for the closed-loop system was an intrahepatic sensor (0.96 mm outer diameter) containing three independent resistance thermometers with an accuracy of ±0.005°C. This sensor was inserted under ultrasound guidance and served as the central reference for thermal control. To assess potential temperature gradients and localized heating effects, additional sensors were positioned in the esophagus, rectum, the urinary bladder, and at multiple skin surface sites.

Patients were maintained under deep anesthesia to achieve hemodynamic stability, suppress sympathetic activation, and ensure patient comfort. Anesthesia was induced with propofol and maintained with sevoflurane at concentrations ≤3%, selected for its minimal hepatic metabolism. In the first patient of cohort C, the maintenance agent was switched to propofol in accordance with the hospital’s standard of care.

Following induction, bladder and gastric catheters were inserted per standard of care. Continuous hemodynamic monitoring included arterial blood pressure, cardiac index, stroke volume variation, systemic vascular resistance, and cardiac output. Thromboelastometry was performed at critical WBHT stages to assess coagulation and fibrinolysis, whereas arterial blood gases were analyzed every 30 minutes throughout the WBHT treatment. Normotension and normocapnia were rigorously maintained per established protocols. Intravenous hydration followed a modified regimen tailored from preclinical data, differing significantly from standard of care to optimize fluid balance during WBHT sessions. Continuous cerebral oximetry was also performed.

Chemotherapy regimens for metastatic PDAC were aligned with the NCCN guidelines to ensure adherence to established oncological standards. However, a cautious dose-escalation approach was employed to maximize safety within the clinical trial setting aimed at evaluating combined treatment tolerability. Gemcitabine was administered the day after the WBHT sessions: a half dose (500 mg/m^2^) was given following the second WBHT session and a full standard dose (1,000 mg/m^2^) was administered the day after the third session. Consequently, the cumulative chemotherapy exposure during the study was lower than that of typical NCCN regimens, with the aim of reducing overall treatment-related toxicity without compromising clinical relevance.

In the first patient enrolled in cohort C, the chemotherapy regimen was modified by replacing gemcitabine monotherapy with a gemcitabine–oxaliplatin combination (GemOx). To maintain consistency with prior cohorts, all subsequent patients in cohort C were treated with the original gemcitabine monotherapy protocol.

### Patient population

The trial was conducted in accordance with the Declaration of Helsinki and the International Conference on Harmonization Good Clinical Practice (ICH-GCP) guidelines and was approved by the Ethical Commission of the University of Antwerp/University Hospital of Antwerp, Belgium. This study was prospectively registered on ClinicalTrials.gov (ID: NCT04467593). Potential candidates were prescreened based on their medical records. Written informed consent was obtained from patients involved in the study.

Eligible patients were adults aged 18 to 75 years at the time of inclusion, diagnosed with either advanced solid tumors (for cohorts A1 and A2) or histologically confirmed metastatic PDAC (for cohorts B and C). Only patients previously treated or under treatment with standard of care were included. Additional inclusion criteria included a World Health Organization performance status of ≤1, a maximum waist circumference of 150 cm, body weight not exceeding 100 kg, and height not exceeding 1.90 m.

Adequate organ function was required, including liver structure suitable for liver sensor placement confirmed by CT, absence of prostate pathology that could interfere with bladder catheter placement, and sufficient bone marrow function defined as white blood cell count ≥2,000/μL, neutrophils ≥1,500/μL, platelets ≥100 × 10^9^/L, and hemoglobin ≥10 g/dL. Coagulation parameters had to meet predefined thresholds: prothrombin time ≥70%, activated partial thromboplastin time within normal limits, von Willebrand factor antigen and activity above the lower limit of normal, and platelet function analyzer closure times within 1.15 times the upper limit of normal (ULN).

Liver function parameters were limited to transaminases [aspartate aminotransferase (AST) and alanine aminotransferase (ALT)] ≤2.5 times ULN, or ≤5 times ULN in the presence of liver metastases, and bilirubin ≤2 times ULN. Renal function requirements included serum creatinine ≤1.6 mg/dL for males and ≤1.3 mg/dL for females, albumin ≥30 g/L, and an estimated glomerular filtration rate ≥60 mL/minute calculated using the Chronic Kidney Disease Epidemiology Collaboration equation, documented within 1 week before inclusion.

Additional eligibility criteria included no blood donation within 3 months prior to WBHT treatment, no participation in other clinical trials, and no biological therapy or chemotherapy within 4 weeks prior or during the WBHT treatment. Surgery was not allowed within 4 weeks before treatment and radiotherapy was prohibited within 3 weeks prior or during treatment. Patients also could not receive antiplatelet agents from 5 days before the first WBHT session until 5 days after the last treatment, or anticoagulants during the study period, except prophylactic low–molecular weight heparin if deemed necessary by investigators, administered from 1 day before the first WBHT session until 10 days after the last session.

Patients were excluded from screening if they met any of the following criteria: pregnancy or breastfeeding, known or suspected brain metastases, or a history of other malignancies within the past 5 years, except for adequately treated basal cell carcinoma or carcinoma *in situ* of the cervix. Additional exclusion criteria included significant comorbidities such as major cardiovascular risk, coronary stenting or myocardial infarction within the previous year, clinically significant pulmonary disease that could interfere with mechanical ventilation, a history of autonomic dysfunction, or malignant hyperthermia (including a relevant family history).

Other exclusion criteria included untreated endocrine disorders (such as uncontrolled diabetes type II or thyroid dysfunction), primary diabetes type I, known allergies to trial medications (including anesthetics, analgesics, or chemotherapy agents used in later cohorts), active infections not controlled by medication, severe nonhealing wounds or fractures, organ transplantation requiring immunosuppression, and any psychiatric or psychosocial disorder impairing compliance or informed consent capability.

### Study design

**Figure 2.**
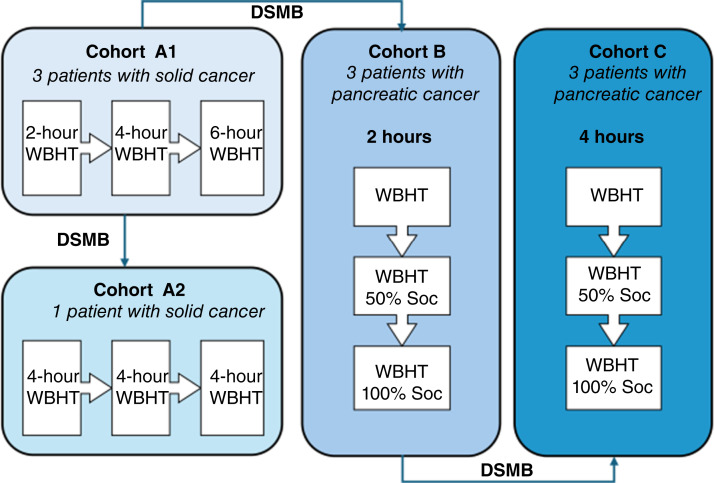
Study design of MATTERS on WBHT dose-escalation and combination treatment in patients with metastatic cancer, with a focus on PDAC. This diagram depicts the MATTERS study, designed to evaluate the safety and tolerability of WBHT in patients with advanced cancer through sequential cohort escalation. In cohort A1, three patients with advanced solid tumors received WBHT monotherapy with increasing durations of 2, 4, and 6 hours. Cohort A2 included one patient who received three repeated WBHT sessions at a fixed intermediate duration of 4 hours to confirm safety and tolerability. Cohorts B and C enrolled patients with metastatic PDAC. In cohort B, patients received 2-hour WBHT sessions as monotherapy first and then combined with 50% and 100% doses of gemcitabine-based standard-of-care (SoC) chemotherapy.

This FIH, single-center safety study was designed to evaluate safety and tolerability at escalating WBHT durations at 41.50°C (Fig. 2). WBHT was administered either as monotherapy or in combination with gemcitabine. The study included four sequential cohorts. The initial cohorts enrolled patients with various metastatic solid tumors, whereas later cohorts focused specifically on metastatic PDAC:

Cohort A1: Three patients with advanced solid tumors, including but not limited to PDAC, received multiple WBHT sessions with increasing durations (2, 4, and 6 hours) to assess safety and tolerability across escalating treatment times.
Cohort A2: One additional patient with advanced solid cancer received three WBHT sessions of 4-hour duration to further evaluate the safety and tolerability of repeated treatments at a consistent thermal dose.
Cohort B: Three patients with metastatic PDAC received three WBHT sessions, each lasting 2 hours. The first session was administered as monotherapy, whereas the second and the third sessions combined WBHT with gemcitabine chemotherapy at 50% (second session) and 100% (third session) of the standard-of-care dosage. This cohort aimed to assess the safety and tolerability of conservative WBHT exposure combined with chemotherapy.
Cohort C: This cohort was designed to mirror cohort B, with patients with PDAC receiving three 4-hour WBHT sessions, to evaluate the safety and tolerability of a higher thermal dose.

A Data Safety Monitoring Board (DSMB) was established to oversee patient safety. After each cohort’s completion, the DSMB conducted a risk assessment using all available safety data before approving progression to the next cohort.

### Outcome measures

The primary objective of this study was to demonstrate technical feasibility and to evaluate the safety and tolerability of WBHT using the TempoCure device for different treatment durations (2, 4, and 6 hours). These durations were tested either as monotherapy or in combination with standard-of-care chemotherapy administered in a fixed schedule of three sessions at 2-week intervals.

To assess safety and tolerability, patients were followed for up to 10 weeks from the last administration of WBHT. Safety assessments were conducted 1 week after the first and the second WBHT sessions. Following the third and last WBHT session, patients attended four additional follow-up visits at approximately weekly intervals, concluding with an end-of-study visit around week 13 to 14.

Adverse events (AE) were recorded throughout the study and graded according to the Common Terminology Criteria for Adverse Events (CTCAE) version 5.0, which classifies AEs from grade 1 (mild) to grade 5 (death). The DSMB prioritized grade ≥3 events for additional review. Serious AEs (SAE) were defined according to ICH-GCP guidelines as any event resulting in death, life-threatening conditions, hospitalization or its prolongation, significant or persistent disability/incapacity, or other medically relevant conditions as determined by the investigators. Causality assessments were performed by the principal investigators, following standard clinical trial procedures, and based on clinical judgment, temporality, biological plausibility, and available literature. The (potential) relationship of each AE to the investigational device or procedure was assessed using a predefined likelihood scale, distinguishing between unrelated events and related events with varying degrees of likelihood.

In addition to standard AE reporting, structured safety monitoring included repeated assessments of vital signs during the procedure and thereafter with detailed physical examinations, comprehensive laboratory testing, continuous cardiac and neurologic monitoring, and frequent coagulation profiling using rotational thromboelastometry. Any results outside normal ranges were reviewed by the investigator and reported as AEs only if considered clinically significant. This safety strategy reflects the heightened vigilance applied because of the novel WBHT technology and the possibility of treatment-related effects being distinct from those seen with conventional chemotherapy. Tolerability was assessed separately, which was defined as the patient’s ability to complete WBHT alone or in combination with chemotherapy without requiring dose modification, treatment interruption, or early termination.

Together, these safety and tolerability assessments provided a comprehensive evaluation of the clinical risk profile of WBHT in this FIH setting. Secondary endpoints assessing preliminary efficacy and quality of life were measured but are not addressed in this safety and tolerability report.

## Results

### Patient baseline characteristics and treatment history

Eligible patients were enrolled after providing written informed consent. Of the 16 individuals recruited across four cohorts, one withdrew prior to screening, and three were excluded because of ineligibility identified during screening. The remaining 12 patients, all with metastatic solid cancer, received treatment between July 2021 and October 2024. The study faced multiple interruptions on request of the hospital during the COVID-19 pandemic.

Patients in cohorts A1 and A2 were out of available treatment options whereas patients in cohorts B and C were in second- or third-line treatment, with some having permanently stopped their chemotherapy treatment. Baseline characteristics of the treated population, all of Caucasian ethnicity, are summarized in Table 1.

**Table 1.** Patient demographics, clinical status, prior treatments (not chronologically sorted), cohort assignment, and screening outcomes for participants enrolled or screened in the MATTERS study.

Patient ID	Sex	Age (years)	Pathology	WHO status	Previous treatments	Recruited for cohort	Est. time from diagnosis to screening (days)	Screening outcome
01	F	47	Cholangiocarcinoma	1	Gemcitabine, cisplatin, FOLFOX, capecitabine, nivolumab, and regorafenib	A1	657	Included
02	M	61	Pseudomyxoma peritonei	0	FOLFIRI	A1	1,004	Included
03	M	63	PDAC	1	Gemcitabine, FOLFIRINOX, FOLFIRI, FOLFOX, 5-FU, leucovorin, and regorafenib	A1	1,146	Included
04	F	51	—	0	None	A1	—	Screen failure
05	M	62	Cholangiocarcinoma	0	Gemcitabine, cisplatin, and capecitabine	A1	248	Included
06	M	58	—	1	None	B	—	Screen failure
07	F	62	PDAC	0	Gemcitabine, abraxane, cisplatin, FOLFIRINOX, nivolumab, and nal-irinotecan	B	1,403	Included
08	M	62	Prostate carcinoma	0	Radiotherapy	A2	700	Included
09	F	52	—	1	None	B	—	No screening
10	F	51	PDAC	1	FOLFIRI	B	525	Included
11	F	52	PDAC	1	Gemcitabine, abraxane, 5-FU, leucovorin, and nal-irinotecan	B	1,821	Included
12	M	58	PDAC	1	Gemcitabine, cisplatin, and FOLFIRINOX	B	702	Included
13	F	58	PDAC	0	Gemcitabine, abraxane, and nal-irinotecan	C	861	Included
14	F	65	—	—	None	C	—	Screen failure
15	F	71	PDAC	0	Gemcitabine, abraxane, and FOLFOX	C	260	Included
16	M	59	PDAC	0	Gemcitabine, abraxane, and FOLFIRINOX	C	407	Included

Abbreviations: 5-FU, 5-fluorouracil; F, female; M, male; WHO, World Health Organization.

WHO status refers to baseline performance status; “Est. time from diagnosis to screening” indicates the approximate days elapsed between initial diagnosis and study screening.

The cohorts included patients diagnosed primarily with disseminated PDAC, along with disseminated cholangiocarcinoma, pseudomyxoma peritonei, and prostate carcinoma. The median age was 59 years (range, 47–71 years), with a balanced distribution between male and female participants. Prior to enrollment, patients had undergone various treatments, as listed in Table 1.

### Tolerability

As summarized in Table 2, a total of 12 patients received a mean of 2.6 WBHT treatments, ranging from one to three sessions, administered as monotherapy or in combination with chemotherapy. Nine patients completed all scheduled WBHT sessions per protocol without the need for any dose modifications or early termination, indicating good adherence and demonstrating the feasibility of repeated WBHT administration within the trial’s framework. Importantly, no treatment was halted prematurely because of device-related or procedural complications, underlining the tolerability and operational practicality of the protocol.

**Table 2.** Overview of WBHT session completion per patient, including details on nonadherence and discontinuation from the MATTERS study.

Patient ID	# WBHT sessions	Treatment completion	Description of (non-)adherence to protocol
01	1	No	Withdrawal due to treatment-related AE, which triggered device improvements
02	3	Yes	Completed study per protocol
03	2	No	Deceased after second treatment because of complications of the underlying disease
05	3	Yes	Deceased shortly before the end-of-study visit
07	3	Yes	Withdrawal shortly before the end-of-study visit
08	3	Yes	Withdrawal shortly before third follow-up visit
10	3	Yes	Completed study per protocol
11	1	No	Withdrawal after first treatment for personal reasons
12	3	Yes	Completed study per protocol
13	3	Yes	Completed study per protocol
15	3	Yes	Completed study per protocol
16	3	Yes	Deceased after third treatment because of disease progression

Data indicate high tolerability of the WBHT regimen, alone or with chemotherapy, in patients with metastatic malignancies.

Three patients did not complete the three scheduled treatments. The first patient (ID 01) discontinued after a single session because of a treatment- and device-related AE, specifically the development of pressure sores at the shoulders, lower back, and heels. In response, modifications were made to the patient installation and positioning, with enforced antidecubitus measures. This resulted in a marked improvement in patient comfort and overall treatment tolerability. The second patient (ID 03) died before receiving the third treatment session. This patient had known portal hypertension at baseline and was admitted to the emergency department 7 days after the second WBHT session with a ruptured esophageal varix. During emergency management, aspiration of blood occurred, leading to respiratory failure. The patient was transferred to the intensive care unit and died 2 days later from related complications. A third patient (ID 11) withdrew consent after the first session for unspecified personal reasons. Although these discontinuations underline the clinical complexity and fragility of patients with advanced metastatic cancer, they do not detract from the demonstrated feasibility and tolerability of the treatment protocol.

### Safety

AEs were reported across all cohorts, with every patient experiencing at least one AE. In addition to AE reporting, the study included comprehensive safety monitoring, comprising serial evaluations of ECG, vital signs, physical examination findings, and routine laboratory assessments, covering hematologic, biochemical, coagulation, and renal function markers. Several changes in blood parameters were observed and classified as AEs. Minor deviations from reference values were observed but not considered clinically relevant.

The principal investigator carefully assessed the relationship of each AE to the investigational device (TempoCure) and/or WBHT, either alone or in combination with chemotherapy. As shown in Supplementary Table S2, a total of 127 AEs were related, to some extent, to the device or treatment. Among these, 14 events (11%) were classified as grade ≥3 in severity according to CTCAE v5.0. Most related AEs were mild to moderate, with 113 events (89%) graded as grade 1 or 2. One related event (0.8%) was grade 4, and one grade 5 event (0.8%) was considered related. A total of nine events (7.1%) met the criteria for SAEs (see Table 3).

**Table 3.** Overview of SAEs reported during the study and assessed as related to TempoCure, WBHT, or their combination, categorized by severity (CTCAE v5.0).

Description of SAEs	Grade 1	Grade 2	Grade 3	Grade 4	Grade 5
Total *n*	1	1	5	1	1
Diarrhea	​	1	​	​	​
Esophageal varix hemorrhage	​	​	​	​	1
Fatigue	​	​	1	​	​
Fever	1	​	​	​	​
Increased creatine kinase	​	​	​	1	​
Malaise	​	​	1	​	​
Obstructive icterus	​	​	1	​	​
RCVS	​	​	1	​	​
Urinary tract infection	​	​	1	​	​

Although SAEs were infrequent, one notable case warrants specific attention. The first patient enrolled in cohort C (ID 13) developed reversible cerebral vasoconstriction syndrome (RCVS) immediately following the third session of WBHT. The initial clinical presentation included confusion and cognitive disorder, progressing to motor aphasia in the absence of other motor deficits. The patient achieved complete clinical recovery within a few hours and was discharged without recurrence during the subsequent 10-week follow-up period.

For cohort C, adjustments were made within the flexibility of the study protocol to optimize both the anesthesia procedure and the chemotherapy regimen. The anesthetic maintenance agent was changed from sevoflurane to propofol, in accordance with the hospital’s standard of care. In parallel, the chemotherapy regimen was modified from gemcitabine monotherapy to GemOx, based on literature suggesting a synergistic effect between WBHT and oxaliplatin.

Following the occurrence of RCVS as SAE, a detailed scientific review was conducted by both the clinical study site (University Hospital Antwerp) and the sponsor (ElmediX). Based on this evaluation, the principal investigator identified the 100% standard-of-care dosage of oxaliplatin as the most likely contributing factor. As a precautionary measure, oxaliplatin was discontinued, and both the original chemotherapy regimen (gemcitabine monotherapy) and anesthesia protocol (sevoflurane maintenance) were reinstated. This isolated SAE underlines the importance of continued vigilance and rigorous safety monitoring as WBHT is further investigated in clinical trials.

To provide insights into commonly occurring events, the most frequently observed AEs, defined as those reported in at least three patients, are summarized in Table 4. Fatigue was the most common, including instances of grade 3 severity. Most fatigue events occurred directly after a WBHT session and gradually disappeared before the following treatment. All affected patients completed the planned treatments without interruption, and fatigue was managed conservatively without the need for treatment modifications or prolonged hospitalization. In most cases, symptoms resolved spontaneously within a few days.

**Table 4.** AEs assessed as related to TempoCure, WBHT, or their combination and reported in three or more patients.

Description of AEs	Total frequency	Grade 1	Grade 2	Grade 3
Fatigue (and fatigue intermittent)	15	3	10	2
Hypokalemia	11	6	5	0
Nausea (and nausea intermittent)	10	8	2	0
Pressure sores	7	4	3	0
Hypotension	7	1	6	0
Anemia	4	0	2	2
Diarrhea	4	3	1	0
Myalgia	5	2	2	1
Hypocalcemia	3	1	2	0
Urinary tract infection	3	0	2	1

Events are sorted by total frequency and categorized by CTCAE v5.0 grade. The majority were grade 1 or 2, with six events classified as grade 3. No grade 4 or 5 related events were observed in three or more patients.

Hypokalemia was also common, with all cases manifesting during or immediately after WBHT. Although serum potassium levels declined transiently, no patient experienced cardiac arrhythmia or other clinical related events. All hypokalemia events were managed with oral or intravenous potassium supplementation per standard hospital protocol, and no cases led to treatment delay, modification, or prolonged hospitalization.

Nausea and diarrhea have been reported in a substantial number of patients, with most events occurring during the treatment period. Both were generally mild to moderate in severity and responded well to supportive care. None of the patients required antiemetic premedication beyond routine use.

Seven cases of pressure sores were documented, most of which occurred during the initial cohorts. These events were primarily low grade and resolved with conservative management. In response, several preventive modifications were introduced, including the use of an antidecubitus mattress with slow foam and intermittent pneumatic (leg) compression devices, along with precise patient positioning to minimize pressure accumulation during treatment. Following these changes, both the frequency and severity of pressure-related events decreased significantly, until they were no longer seen in the latter cohorts of the study.

Four cases of treatment-related anemia were reported. Observed low bilirubin levels pointed toward a transient dilutional effect, likely caused by intraprocedural fluid shifts and volume loading, rather than hemolysis as the primary contributing factor.

Three cases of urinary tract infection were classified as related to treatment, most likely attributable to the use of an indwelling catheter.

No device malfunctions or operational issues occurred during the study. Additionally, there were no treatment-related deaths or discontinuations due to device-related AEs.

Although formal statistical analysis was not feasible because of the small sample size, a descriptive evaluation of related AE and SAE occurrence related to WBHT administered alone or in combination with chemotherapy was conducted and is summarized in Table 5. The influence of WBHT treatment duration on the frequency of related AEs and SAEs was also examined, with findings presented in Table 6.

**Table 5.** Incidence of related AEs and SAEs observed during WBHT administered as monotherapy or in combination with chemotherapy.

Treatment	Number of treatments	Total AE	Total SAE	Total AE + SAE	AE per treatment	SAE per treatment	AE + SAE per treatment
WBHT	19	80	8	88	4.21	0.42	4.63
WBHT + chemotherapy	12	38	1	39	3.17	0.08	3.25

Data are presented as total events and mean events per session.

**Table 6.** Distribution of related AEs and SAEs according to the duration of WBHT treatments.

WBHT duration	Number of treatments (*n*)	Total AE	Total SAE	Total AE + SAE	AE per treatment	SAE per treatment	AE + SAE per treatment
2 hours	14	56	2	58	4	0.14	4.14
4 hours	15	56	5	61	3.73	0.33	4.07
6 hours	2	6	2	8	3	1	4

Data include total events and mean events per treatment session for 2-hour, 4-hour, and 6-hour WBHT exposures.

No clear differences in the incidence of related AEs or SAEs were observed between WBHT monotherapy and combination therapy with chemotherapy. There was a trend toward fewer related AEs in the combination therapy group, which corresponded with later study cohorts and may reflect the impact of a learning curve and procedural improvements.

With regard to treatment duration, no increase in related AEs was observed with longer WBHT sessions. A slight trend toward fewer related AEs but a marginal increase in SAEs was noted in the 6-hour treatments; however, the limited sample size prevents definitive conclusions. These longer sessions also occurred later in the study, consistent with potential learning curve effects.

Overall, these findings support an acceptable safety profile for WBHT in both monotherapy and combination with chemotherapy, with no obvious worsening of AEs related to longer treatment durations.

## Discussion

This FIH study evaluated the safety, tolerability, and procedural feasibility of WBHT delivered via the TempoCure system, both as monotherapy and in combination with chemotherapy, in patients with advanced malignancies, including metastatic PDAC. Overall, WBHT at a fixed target core temperature of 41.50°C was well tolerated, and most patients completed the scheduled treatment regimens despite extensive prior therapies, highlighting the clinical feasibility of this approach in a challenging patient population.

Fatigue emerged as the most frequently reported AE, followed by hypokalemia, nausea, and diarrhea. These events were predominantly mild to moderate in severity and transient, resolving with standard supportive care. The hematologic toxicity profile, including anemia, was consistent with expected chemotherapy effects and showed no evidence of hemolysis attributable to WBHT. Device-related toxicity was minimal. Early occurrences of pressure sores, confined mainly to the first study cohort, reflected the learning curve associated with the novel procedure. Implementation of optimized patient positioning and pressure sore preventive measures markedly reduced these events in later cohorts, approaching near elimination. These findings emphasize that procedural refinements and accumulated experience are vital to minimizing complications when introducing novel medical devices.

Formal statistical comparisons of AEs were limited by the small sample size and study design. The DSMB of this study endorsed a descriptive evaluation that revealed no clear differences in related AE or SAE incidence between WBHT monotherapy and in combination with chemotherapy. A slight trend toward fewer related AEs during combination therapy, administered in later treatment sessions, may reflect a learning curve effect and the benefits of iterative device and procedural improvements. Similarly, evaluation across WBHT durations (2, 4, and 6 hours) revealed no clear association between treatment length and the frequency or severity of related AEs.

Device performance was robust throughout the study, with no malfunctions or operational failures, supporting the technical reliability of the TempoCure system. All procedures were delivered efficiently, without delays, technical issues, or safety compromises, demonstrating that prolonged WBHT sessions can be reliably performed in a controlled clinical setting. Rigorous safety-monitoring protocols, including cerebral and cardiac surveillance and frequent coagulation assessments, were instituted at trial onset given the novelty of this WBHT protocol and device. Although the operating room was used in this study to allow immediate intervention if needed, less resource-intensive environments, such as the postanesthesia care unit or intensive care unit, could be considered for future trials to improve feasibility and cost efficiency.

Continuous temperature monitoring was performed at multiple anatomic sites using TempoSensor flexible probes, including intrahepatic, esophageal, rectal, urinary bladder, and skin surface locations. The intrahepatic measurement served as a reliable surrogate for systemic thermal exposure and approximated the highest expected tumor temperature. Device-based and large-animal models (20) provided translational support for this approach, allowing careful extrapolation of intratumoral heating while maintaining patient safety. Together, these findings demonstrate that WBHT can be delivered safely and efficiently, and they provide a foundation for procedural refinements to optimize treatment delivery and scalability.

Planned procedural innovations for future trials include noninvasive temperature monitoring, alternative anesthetic strategies, and centralized monitoring models akin to dialysis units, which could allow a single specialized team to supervise multiple treatments simultaneously. These strategies aim to simplify WBHT delivery, improve scalability across multiple sites, and ensure broader patient access while maintaining safety and procedural control.

Protocol flexibility was an essential feature of this FIH study. For example, oxaliplatin was administered to one patient in combination with WBHT based on preclinical evidence suggesting potential synergistic effects (22–25), including enhanced cytotoxicity, improved intratumoral drug uptake, and modulation of tumor vasculature. Although this combination demonstrated a manageable safety profile, it may not be broadly applicable. These experiences provide critical guidance for procedural planning and combination strategies in future efficacy-focused studies.

Exploratory signals of biological activity and efficacy were observed. It should be noted that this population was small and heterogeneous, limiting the interpretability of these findings. Several patients with pancreatic cancer exhibited stabilization or decreases in CA19-9 levels during the active treatment phase. Transient activation of immune-related signaling molecules, particularly immune-stimulatory cytokines, was measured. Short-term disease stabilization occurred in three of eight treated patients, with one patient maintaining stable disease at the 14-week assessment. Although preliminary and descriptive, these findings provide valuable insights for refining patient selection, endpoint definitions, and study design for subsequent trials. A more comprehensive analysis of these signals may be reported separately as part of ongoing research.

The MATTERS trial concluded ahead of schedule following recommendations from the principal investigators and approval by the DSMB, reflecting successful achievement of safety objectives. The single-center design facilitated tight control over treatment delivery and safety monitoring, enabling rapid identification and mitigation of early safety signals. To improve generalizability, the upcoming MATTERS-2 trial will be a multicenter, two-arm, randomized controlled pivotal study evaluating WBHT in second-line metastatic pancreatic cancer in approximately 90 adults with Eastern Cooperative Oncology Group performance status of one or less. In MATTERS-2, the experimental arm will receive multiple WBHT sessions at 41.50°C for 4 hours under anesthesia using the TempoCure system in combination with investigator’s choice of standard-of-care chemotherapy, whereas the control arm will receive chemotherapy alone. Primary endpoints will include overall survival and safety and tolerability of repeated WBHT procedures, with secondary endpoints encompassing overall response rate, progression-free survival, quality of life, and CA19-9 evolution. Continuous data safety monitoring will ensure rigorous oversight. The design of MATTERS-2 directly integrates lessons from MATTERS, including procedural refinements, surrogate temperature monitoring, procedural innovations, and optimal treatment delivery. This larger study will provide the statistical power and patient homogeneity necessary to confirm safety and demonstrate efficacy.

Given the dismal prognosis and limited therapeutic options for patients with metastatic PDAC, the development of innovative treatments like WBHT is urgently needed. The MATTERS study was designed to establish safety and technical feasibility using a fixed temperature target of 41.50°C based on translational data demonstrating synergistic effects with chemotherapy and tolerability within physiologic limits. The precise temperature control afforded by the TempoCure medical device is a pivotal advancement, addressing prior challenges related to thermal dose delivery and safety in the HT domain. Achieving optimal thermal dosing within a clinically practical treatment duration is essential for balancing therapeutic benefit and patient safety and tolerability.

The MATTERS trial demonstrated that WBHT at 41.50°C with the TempoCure medical device is safe and well tolerated in patients with advanced malignancies, including metastatic PDAC. The safety profile was characterized by manageable AEs without serious device-related complications. The high patient adherence to the protocol reflected good treatment tolerability. These findings provide a robust foundation for advancing to randomized efficacy trials. Future studies will aim to rigorously assess the clinical efficacy of WBHT in combination with other anticancer treatments in larger patient populations.

## Supplementary Material

Table S1 Representativeness of Study Participants

Table S2 Overview of adverse events (AE) reported during the study and assessed as related to TempoCure, whole-body hyperthermia or their combination, categorized by severity (CTCAE v5.0).

## Data Availability

The clinical data generated in this study are not publicly available because of patient privacy requirements and ethical considerations. However, deidentified data may be made available upon reasonable request from the corresponding author and with approval from the appropriate ethics committee.
