# Supplementary material for: The MATTERS Trial: Safety and Tolerability of Whole-Body Hyperthermia at 41.50°C in Combination with Chemotherapy in Metastatic Cancer Patients
Source: Cancer Res Commun. 2026 Feb 4;6(2):273–83. doi: 10.1158/2767-9764.CRC-25-0660 (PMC12869149; doi:10.1158/2767-9764.CRC-25-0660)
Supplement: Table S1 — Representativeness of Study Participants [file crc-25-0660_table_s1_suppst1.pdf]

Table S1. Representativeness of Study Participants

**Cancer type(s)/stage(s):**<br>Advanced solid tumours, including metastatic pancreatic ductal adenocarcinoma (PDAC)	
**Considerations related to:**	**Details**
**Sex**	Advanced solid tumours, including metastatic PDAC, affect both sexes. Globally men constitute a higher proportion of advanced cancer patients (60%) compared to women (40%) though this varies by cancer type.<br>In this study, the distribution was balanced, with 58% screened (50% included) female and 42% screened (50% included) male patients.
**Age**	For advanced/metastatic solid tumours, median ages in published series typically range from 59 to 66 years with median ages typically above 65 years for PDAC. The median age of screened participants was 59 years (range: 47–71 years).
**Race/Ethnicity**	All participants in the MATTERS trial were Caucasian, reflecting the demographics of the trial site in Belgium. This limits the representativeness of the study for other racial/ethnic groups, particularly Black, Hispanic, and Asian populations.
**Geography**	The study was conducted at a single centre in Antwerp, Belgium, with patients recruited locally. This geographic focus may limit applicability to broader populations, particularly those outside of European settings.
**Other Considerations**	Patients included in the study had advanced malignancies and were heavily pretreated with chemotherapy regimens. The trial enrolled patients with good functional status (WHO performance status 0–1) and excluded those with significant comorbidities or organ dysfunction. While this selection is consistent with standard clinical trial criteria, it does not account for the smaller proportion of real-world patients (~30%) who may present with poorer performance status.
**Overall Representativeness of the Study**	The study population reflects the demographics of advanced cancer patients in a European setting but excludes racial/ethnic diversity and patients with poorer baseline health. The findings are applicable to heavily pretreated advanced cancer patients but may not generalize to populations with different genetic, environmental, or socioeconomic backgrounds.
