# Supplementary material for: The MATTERS Trial: Safety and Tolerability of Whole-Body Hyperthermia at 41.50°C in Combination with Chemotherapy in Metastatic Cancer Patients
Source: Cancer Res Commun. 2026 Feb 4;6(2):273–83. doi: 10.1158/2767-9764.CRC-25-0660 (PMC12869149; doi:10.1158/2767-9764.CRC-25-0660)
Supplement: Table S2 — Overview of adverse events (AE) reported during the study and assessed as related to TempoCure, whole-body hyperthermia or their combination, categorized by severity (CTCAE v5.0). [file crc-25-0660_table_s2_suppst2.docx]

**Table S2: Overview of adverse events (AE) reported during the study and assessed as related to TempoCure®, whole-body hyperthermia or their combination, categorized by severity (CTCAE v5.0).**

| **Adverse Event** | | **Grade 1** | **Grade 2** | **Grade 3** | **Grade 4** | **Grade 5** | **Total** |
| --- | --- | --- | --- | --- | --- | --- | --- |
| **Total n.** | | **63** | **50** | **12** | **1** | **1** | **127** |
| **Systemic and Metabolic** | | **18** | **20** | **5** | **1** | **0** | **44** |
|  | Anemia | 0 | 2 | 2 | 0 | 0 | 4 |
|  | Anorexia | 1 | 0 | 0 | 0 | 0 | 1 |
|  | Blood lactate dehydrogenase increased | 1 | 0 | 0 | 0 | 0 | 1 |
|  | Fatigue | 2 | 7 | 2 | 0 | 0 | 11 |
|  | Fatigue intermittent | 1 | 3 | 0 | 0 | 0 | 4 |
|  | Fever | 2 | 0 | 0 | 0 | 0 | 2 |
|  | Hypocalcemia | 1 | 2 | 0 | 0 | 0 | 3 |
|  | Hypokalemia | 6 | 5 | 0 | 0 | 0 | 11 |
|  | Hypomagnesemia | 1 | 0 | 0 | 0 | 0 | 1 |
|  | Increased CK | 0 | 0 | 0 | 1 | 0 | 1 |
|  | Increased creatinine clearance | 1 | 0 | 0 | 0 | 0 | 1 |
|  | Increased lactate | 0 | 1 | 0 | 0 | 0 | 1 |
|  | Malaise | 0 | 0 | 1 | 0 | 0 | 1 |
|  | Weight loss | 2 | 0 | 0 | 0 | 0 | 2 |
| **Dermatologic / Mucosal** | | **15** | **7** | **0** | **0** | **0** | **22** |
|  | Allergic reaction on patches | 1 | 0 | 0 | 0 | 0 | 1 |
|  | Bedsore heels | 0 | 1 | 0 | 0 | 0 | 1 |
|  | Blister left elbow outside | 1 | 0 | 0 | 0 | 0 | 1 |
|  | Blister on back of head | 1 | 0 | 0 | 0 | 0 | 1 |
|  | Blister thorax | 1 | 0 | 0 | 0 | 0 | 1 |
|  | Blisters right middle finger | 0 | 1 | 0 | 0 | 0 | 1 |
|  | Blisters thumb & index fingers | 2 | 0 | 0 | 0 | 0 | 2 |
|  | Burning feeling lips | 1 | 0 | 0 | 0 | 0 | 1 |
|  | Dry skin | 1 | 0 | 0 | 0 | 0 | 1 |
|  | Herpes labialis | 1 | 1 | 0 | 0 | 0 | 2 |
|  | Pressure sores | 3 | 2 | 0 | 0 | 0 | 5 |
|  | Pressure sores right hip | 1 | 0 | 0 | 0 | 0 | 1 |
|  | Pressure sores tailbone | 0 | 1 | 0 | 0 | 0 | 1 |
|  | Redness right elbow | 1 | 0 | 0 | 0 | 0 | 1 |
|  | Swollen eyelids | 0 | 1 | 0 | 0 | 0 | 1 |
|  | Wound right corner in mouth | 1 | 0 | 0 | 0 | 0 | 1 |
| **Gastrointestinal** | | **13** | **6** | **2** | **0** | **0** | **21** |
|  | Abdominal pain | 0 | 1 | 0 | 0 | 0 | 1 |
|  | Diarrhea | 2 | 1 | 0 | 0 | 0 | 3 |
|  | Diarrhea intermittent | 1 | 0 | 0 | 0 | 0 | 1 |
|  | Gastritis | 0 | 1 | 0 | 0 | 0 | 1 |
|  | Nausea | 1 | 1 | 0 | 0 | 0 | 2 |
|  | Nausea intermittent | 7 | 1 | 0 | 0 | 0 | 8 |
|  | Obstructive icterus | 0 | 0 | 1 | 0 | 0 | 1 |
|  | Oral mucositis | 1 | 1 | 0 | 0 | 0 | 2 |
|  | Pain right hypochonder | 0 | 0 | 1 | 0 | 0 | 1 |
|  | Vomiting | 2 | 0 | 0 | 0 | 0 | 2 |
|  | Vomiting intermittent | 1 | 0 | 0 | 0 | 0 | 1 |
| **Cardiovascular** | | **4** | **8** | **1** | **0** | **1** | **14** |
|  | Abscess vena jugularis | 0 | 1 | 0 | 0 | 0 | 1 |
|  | Edema fingers and arms | 1 | 0 | 0 | 0 | 0 | 1 |
|  | Edema lower limbs | 1 | 0 | 0 | 0 | 0 | 1 |
|  | Epistaxis | 1 | 0 | 0 | 0 | 0 | 1 |
|  | Esophageal varices hemorrhage | 0 | 0 | 0 | 0 | 1 | 1 |
|  | Hypertension | 0 | 0 | 1 | 0 | 0 | 1 |
|  | Hypotension | 1 | 6 | 0 | 0 | 0 | 7 |
|  | Thrombophlebitis right arm | 0 | 1 | 0 | 0 | 0 | 1 |
| **Neurological** | | **5** | **4** | **1** | **0** | **0** | **10** |
|  | Amnesia | 0 | 1 | 0 | 0 | 0 | 1 |
|  | Confusion | 2 | 0 | 0 | 0 | 0 | 2 |
|  | Dizziness | 2 | 0 | 0 | 0 | 0 | 2 |
|  | Foot drop left | 0 | 2 | 0 | 0 | 0 | 2 |
|  | Foot drop right | 0 | 1 | 0 | 0 | 0 | 1 |
|  | Headache intermittent | 1 | 0 | 0 | 0 | 0 | 1 |
|  | Reversible Cerebral vasoconstriction syndrome | 0 | 0 | 1 | 0 | 0 | 1 |
| **Musculoskeletal** | | **4** | **3** | **2** | **0** | **0** | **9** |
|  | Back pain | 0 | 1 | 0 | 0 | 0 | 1 |
|  | Muscle cramp intermittent | 1 | 0 | 0 | 0 | 0 | 1 |
|  | Muscle weakness lower left limb | 1 | 0 | 1 | 0 | 0 | 2 |
|  | Myalgia | 2 | 2 | 0 | 0 | 0 | 4 |
|  | Myalgia both elbows | 0 | 0 | 1 | 0 | 0 | 1 |
| **Urinary System** | | **1** | **2** | **1** | **0** | **0** | **4** |
|  | Dysuria | 1 | 0 | 0 | 0 | 0 | 1 |
|  | Urinary Tract Infection | 0 | 2 | 1 | 0 | 0 | 3 |
| **Other** | | **1** | **0** | **0** | **0** | **0** | **1** |
|  | Voice alterations | 1 | 0 | 0 | 0 | 0 | 1 |
